# Sonographic findings of COVID‐Arm: A case series

**DOI:** 10.1002/jvc2.49

**Published:** 2022-10-18

**Authors:** Priscila Giavedoni, Alba Català Gonzalo, Sara Gómez‐Armayones

**Affiliations:** ^1^ Servicio de Dermatología. Hospital Clínic Barcelona Spain

**Keywords:** COVID‐19, COVID‐Arm, dermatology, high‐frequency‐ultrasound, SARS‐CoV‐2, vaccines

## INTRODUCTION

Erythematous patches or swollen plaques appearing usually after 4 days or more at the injection site of SARS‐CoV‐2 vaccination are commonly known as ‘COVID‐Arm’.^1^ It is considered normal to have signs of inflammation up to 3 days after the vaccine is administered. Pathological inflammatory reactions are those that persist beyond 3 days. COVID‐Arm is one of the most commonly reported adverse effect of all COVID‐19 vaccines, but it seems much more frequent with mRNA‐1273.^2^, ^3^, ^4^ COVID‐Arm is almost exclusively described in women.^5^ Skin biopsies in COVID‐Arm have shown superficial perivascular and perifollicular lymphocytic infiltrates, with rare eosinophils and scattered mast cells. These findings correspond with a delayed‐type hypersensitivity reaction.^6^

High‐frequency ultrasound (HFUS)^7^ aids in the diagnosis and follow‐up of inflammatory skin diseases.^8^ We describe herein the ultrasound characteristics of local injection‐site cutaneous adverse reactions after vaccination against SARS‐CoV‐2.

## PATIENTS

We studied seven patients with COVID‐Arm attended in our department between the 1st of March and the 30th of June, 2021. All patients were Caucasian females, with age between 27 and 59 years (median, 37 years). We excluded patients with injection‐site reactions lasting ≤3 days, as this reaction was very common in SARS‐CoV‐2 vaccine randomised controlled trials.^9^

The study was authorised by the Ethics Committees of our institution. All patients gave written informed consent to participate and explicit consent to publish images.

## TECHNIQUE

HFUS was performed by a dermatologist trained in the diagnosis and management of autoimmune diseases and HFUS. Esaote MyLab™Class C equipment was used with high‐frequency probes between 10, 18 and 22 MHz.

The following cut‐off values were used to determine negative Doppler: isolated vessels in the dermis and hypodermis, with a systolic peak of <10 cm/s and a resistance index of <0.7.^2^, ^10^ Active panniculitis was defined as fulfilment of ≥2 of the three criteria in Doppler mode: systolic peak >10 cm/s, resistance index >0.7 MHz, and vessel diameter >1 mm.

The variables analysed in mode B of the HFUS considered to be indicative of inflammation were hypoechogenicity of the dermis, hyperechogenicity of lobes in all cases with or without hypoechogenicity and thickening of the subcutaneous septa.^3^, ^8^ Increased or decreased echogenicity was considered in comparison with healthy perilesional and contralateral skin. Septa were considered thickened if 3 or more septa were thicker than 1 mm.^10^ Other signs present in active disease were hypoechogenicity of the dermis and loss of the dermo‐hypodermal line.^8^

## RESULTS

**Figure 1:**
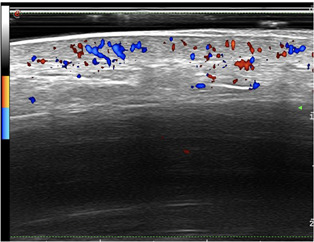
Case 3. Hypoechogenicity and increased Doppler flow in the dermis.

**Figure 2:**
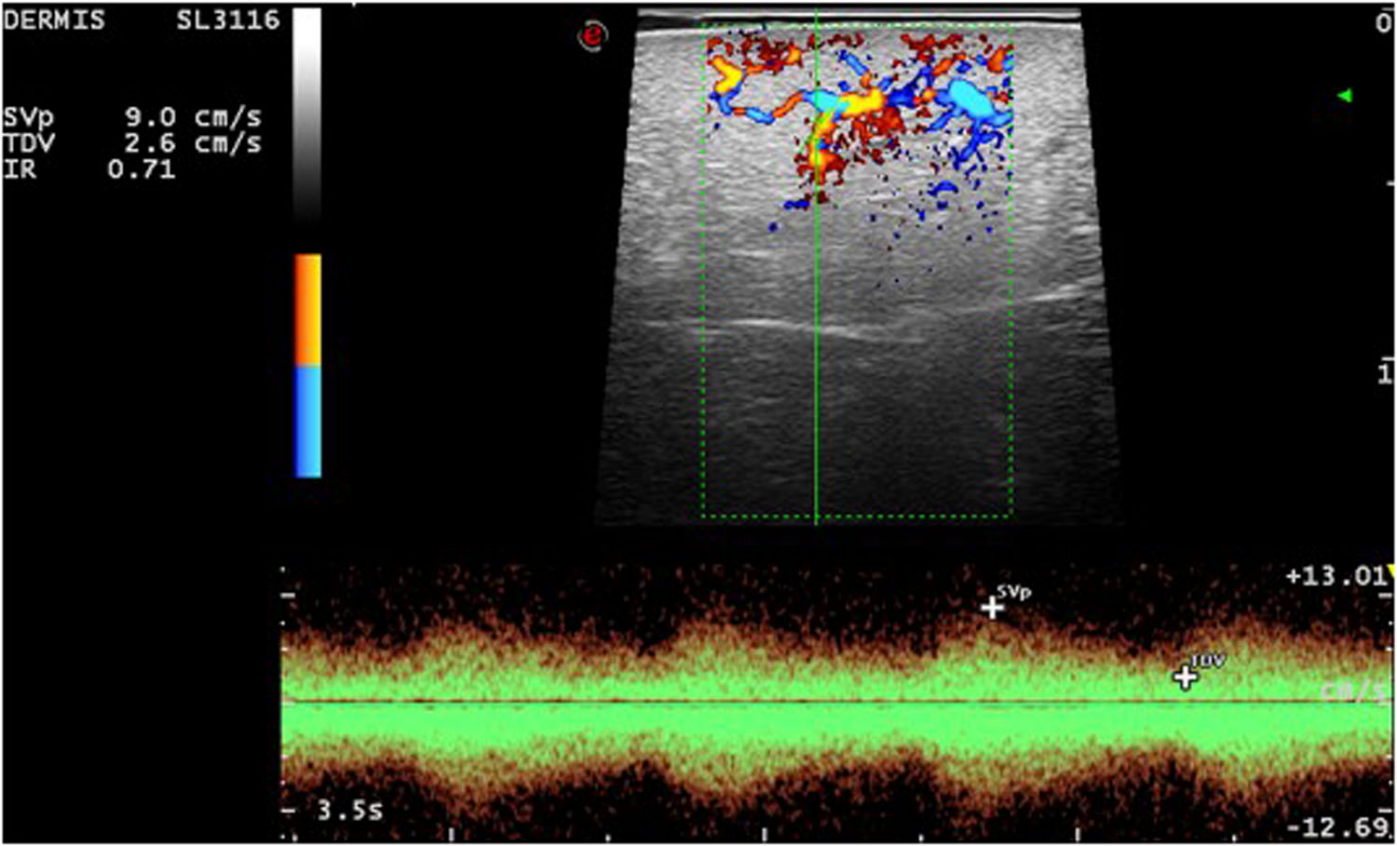
Case 1. Increased vascularisation in dermis and hypodermis. Arterial vessels with a 9 cm/s systolic peak, diastolic 2.6 cm/s, and a resistance index of 0.71.

**Figure 3:**
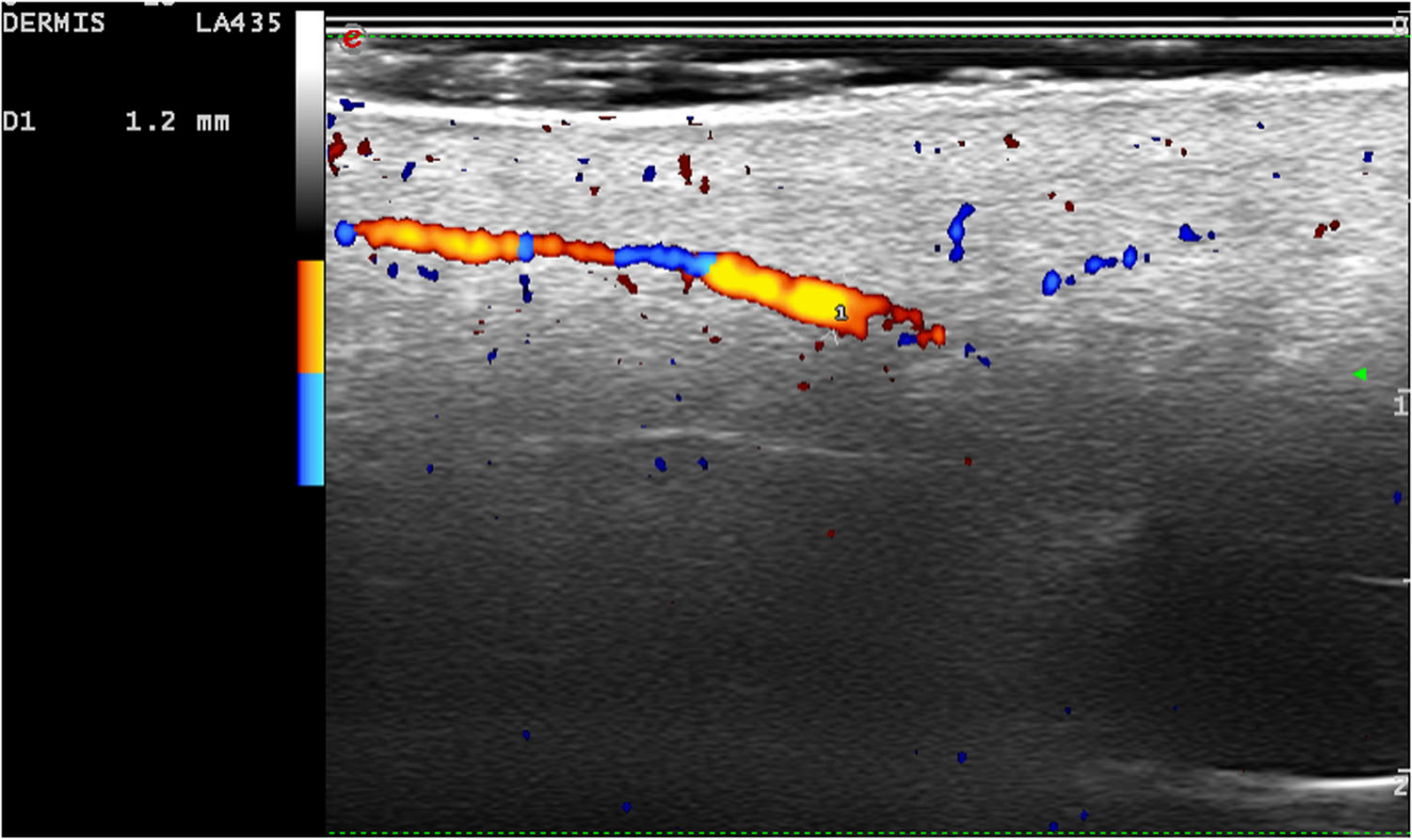
Case 1. Increased vascularisation in the hypodermis, with arterial vessels measuring 1.2 mm in diameter

**Figure 4:**
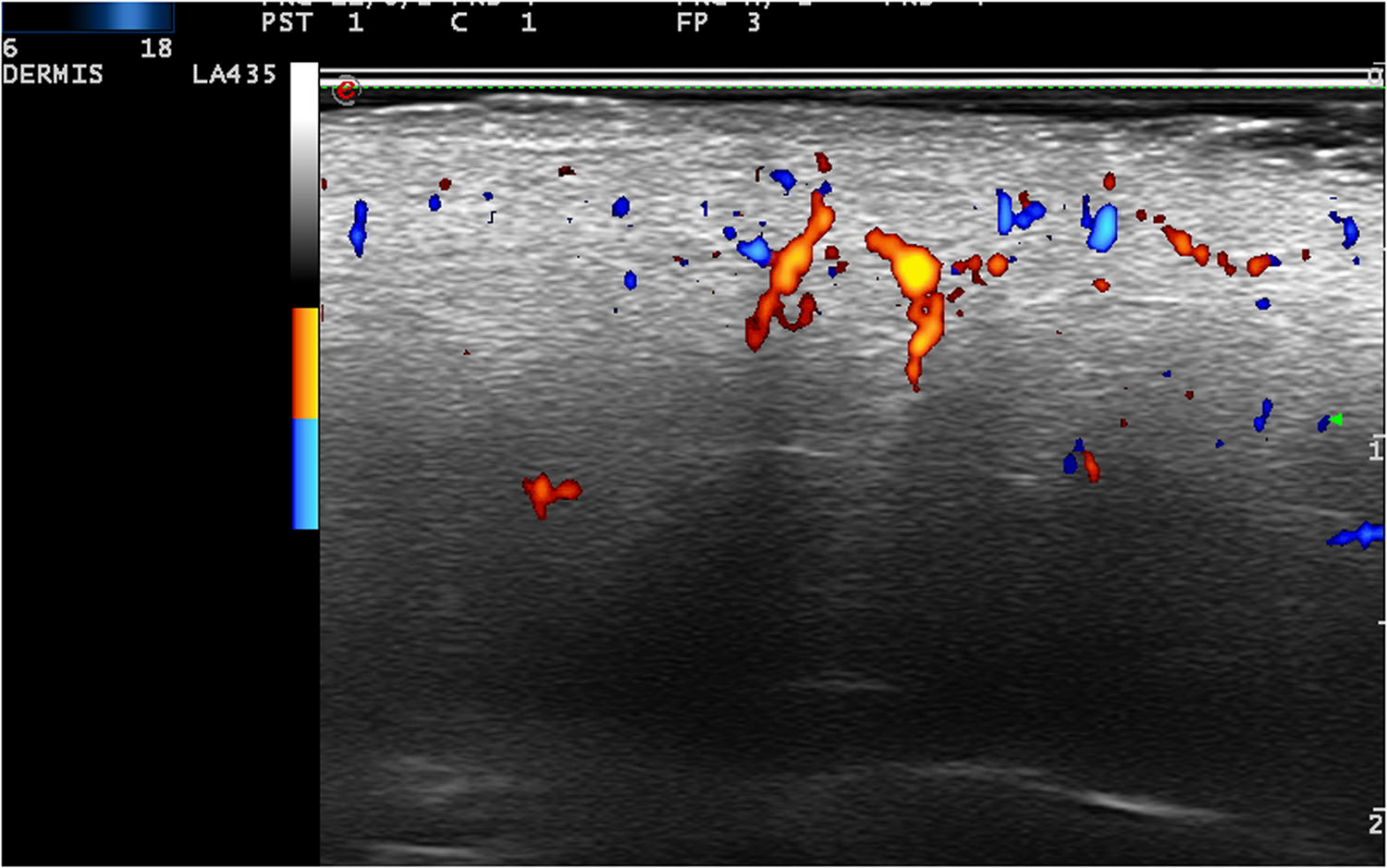
Case 5. Power Doppler Colour: increased vascularisation in dermis and hypodermis.

Table 1 shows the clinical and HFUS characteristics of patients. In one patient, localised inflammation was observed only in the dermis, without signs of panniculitis (Figure 1). Inflammation of the hypodermis was observed in six of the seven patients (85.7%); in five (71.4%) cases, the ultrasound pattern was lobular panniculitis and in only one (14.3%) was mixed panniculitis (septal and lobular). Vessel diameter was increased, with a median of 1.4 mm (IQR: 0.8−1.7 mm), and a median peak systolic velocity of 7 cm/s (IQR: 5−15.2 cm/s) (Figures 2, 3, 4).

**Table 1:** Clinical and high‐frequency ultrasound characteristics of patients

Case	Age	Medical history	Dose	Day of onset	Duration	Systemic symptoms	Treatment	US findings
**1**	37	Depression	2nd (selfreported also for 1st dose)	1	10	Fever	Topical corticosteroids—oral antihistamines	Hypoechoic dermis. Loss of differentiation between dermis and hypodermis. Hyperechogenicity of the lobules and hypoechogenicity of the septa. Doppler with an intense increase of flow in dermis and hypodermis: arterial vessels of 1.2 mm diameter and high velocity compared to healthy skin, with a systolic peak of 9 cm/s and a resistance index of 0.71.
**2**	27	No	1st	4	7	Nausea, diarrhoea, headache painful lymph node	Topical corticosteroids—NSAIDs	Hypoechoic dermis. Loss of differentiation between dermis and hypodermis. Hyperechogenicity of the lobules. Doppler with an intense increase in flow in dermis and hypodermis: arterial vessels of 1.4 mm in diameter and a high velocity compared to healthy skin, with a systolic peak of 7 cm/s and a resistance index of 0.70.
3	37	No	1st	5	10	Painful lymph node	Topical corticosteroids—NSAIDs	Hypoechogenicity of the dermis with an intense increase of flow in Doppler mode, fine arterial vessels of low velocity systolic peak 5 cm/s. Preservation of dermo‐hypodermal differentiationHypodermis of normal appearanceEnlarged lymph node: 20.1 × 9.3 mm, with intense vascularisation in the hilum towards the periphery; with arterial vessels up to 1.2 mm in diameter and a very high velocity: the systolic peak of 37.2 cm/s and resistance index of 0.87.
**4**	59	No	1st	6	8	Headache, fever	Topical corticosteroids—oral antihistamines	Dermis with echogenicity similar to healthy skin. Loss of differentiation between dermis and hypodermis. Hyperechogenicity of the lobules. Doppler with a mild increase of flow in dermis and hypodermis: arterial vessels of 1.2 mm in diameter and low velocity, with a systolic peak of 5 cm/s.
**5**	30	No	1st	8	a	Asthenia	Paracetamol—topical corticosteroids	Hypoechogenicity of the dermis. Loss of differentiation between dermis and hypodermis. Hyperechogenicity of the lobules. Doppler with an intense increase of flow in dermis and hypodermis: arterial vessels of 1.1 mm in diameter and high velocity compared to healthy skin, with a systolic peak of 15.2 cm/s and a resistance index of 0.77.
6	33	Hypothyroidism	1st	1	10	Painful lymph node	No	Dermis with echogenicity similar to healthy skin. Loss of differentiation between dermis and hypodermis. Hyperechogenicity of the lobules. Doppler with slightly increased flow in dermis and hypodermis: arterial vessels of 0.8 mm diameter and low velocity, with a systolic peak of 5 cm/s.
7	52	No	1st	7	14	Headache, myalgia	Topical corticosteroids—oral antihistaminesParacetamol	Dermis with echogenicity similar to healthy skin. Loss of differentiation between dermis and hypodermis. Hyperechogenicity of the lobules. Doppler with the significantly increased flow in dermis and hypodermis: arterial vessels of 1.7 mm in diameter and high velocity, with a systolic peak of 10.6 cm/s and a resistance index of 0.73.

^a^ Data not reported.

**Figure 5:**
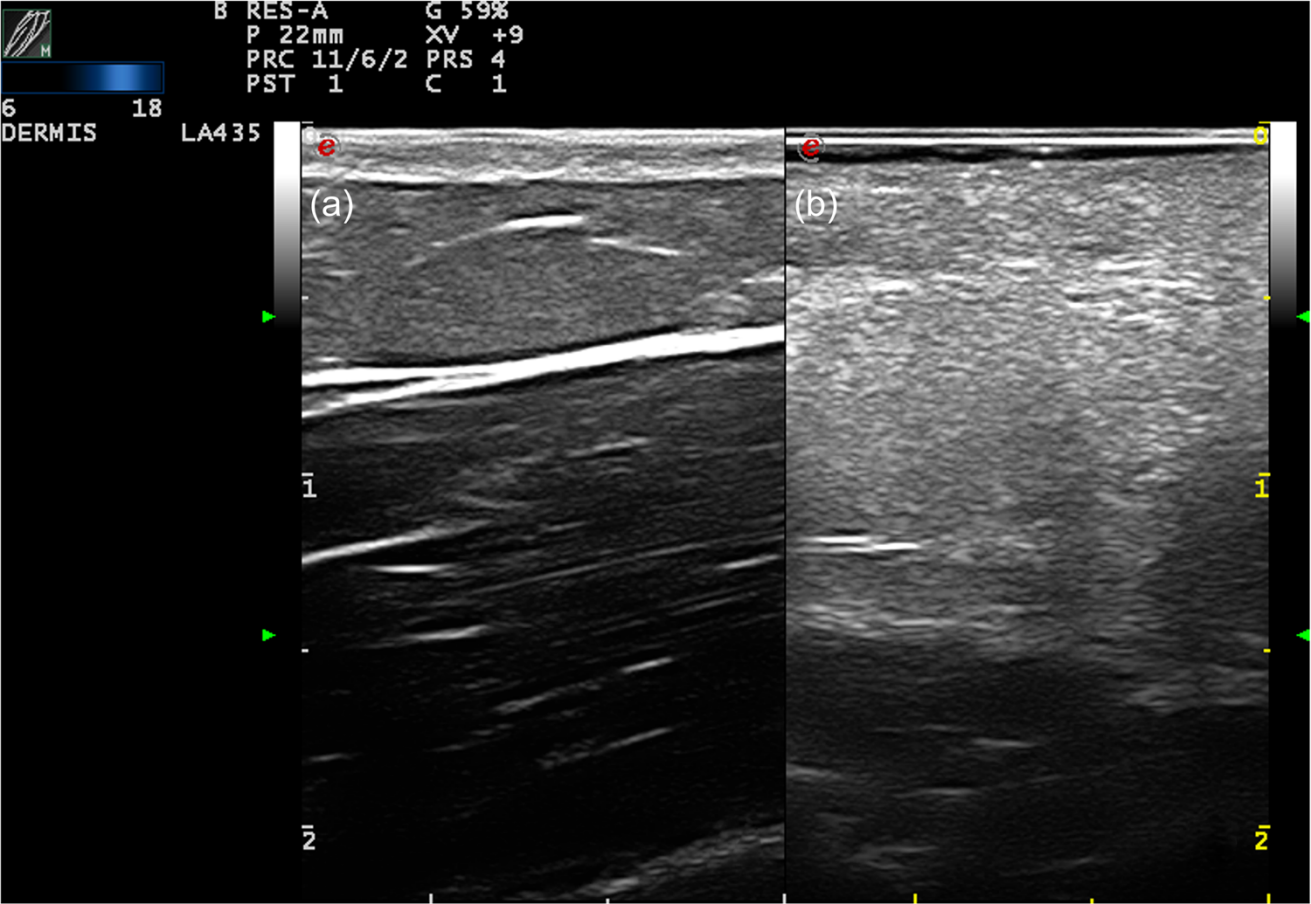
Case 4. (a) Healthy skin. d, dermis; sc, subcutaneous tissue; m, muscle. (b) Panniculitis: hypoechogenicity of the dermis and hyperechogenicity of the lining of the dermis.

**Figure 6:**
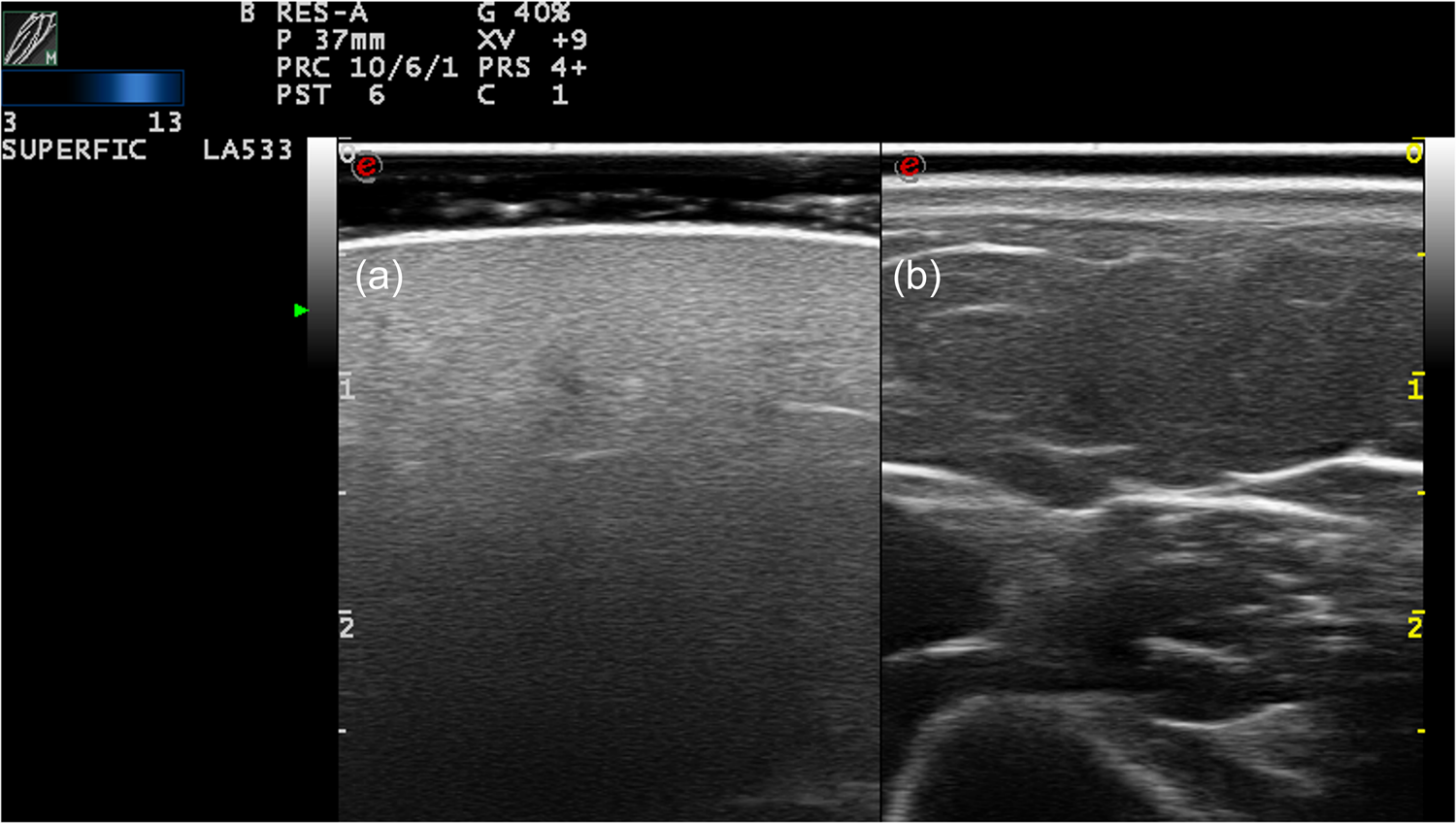
Case 6. (a) Panniculitis: loss of differentiation between dermis and hypodermis. (b) Healthy skin. d, dermis; sc, subcutaneous tissue; m, muscle.

Areas of inflammation were observed with hypoechogenicity of the dermis and hyperechogenicity of lobules in the hypodermis. In addition, in patients with panniculitis, a loss of differentiation between dermis and hypodermis was seen. These findings can be seen in Figures 5 and 6, where the inflamed area is compared with the contralateral healthy skin.

**Figure 7:**
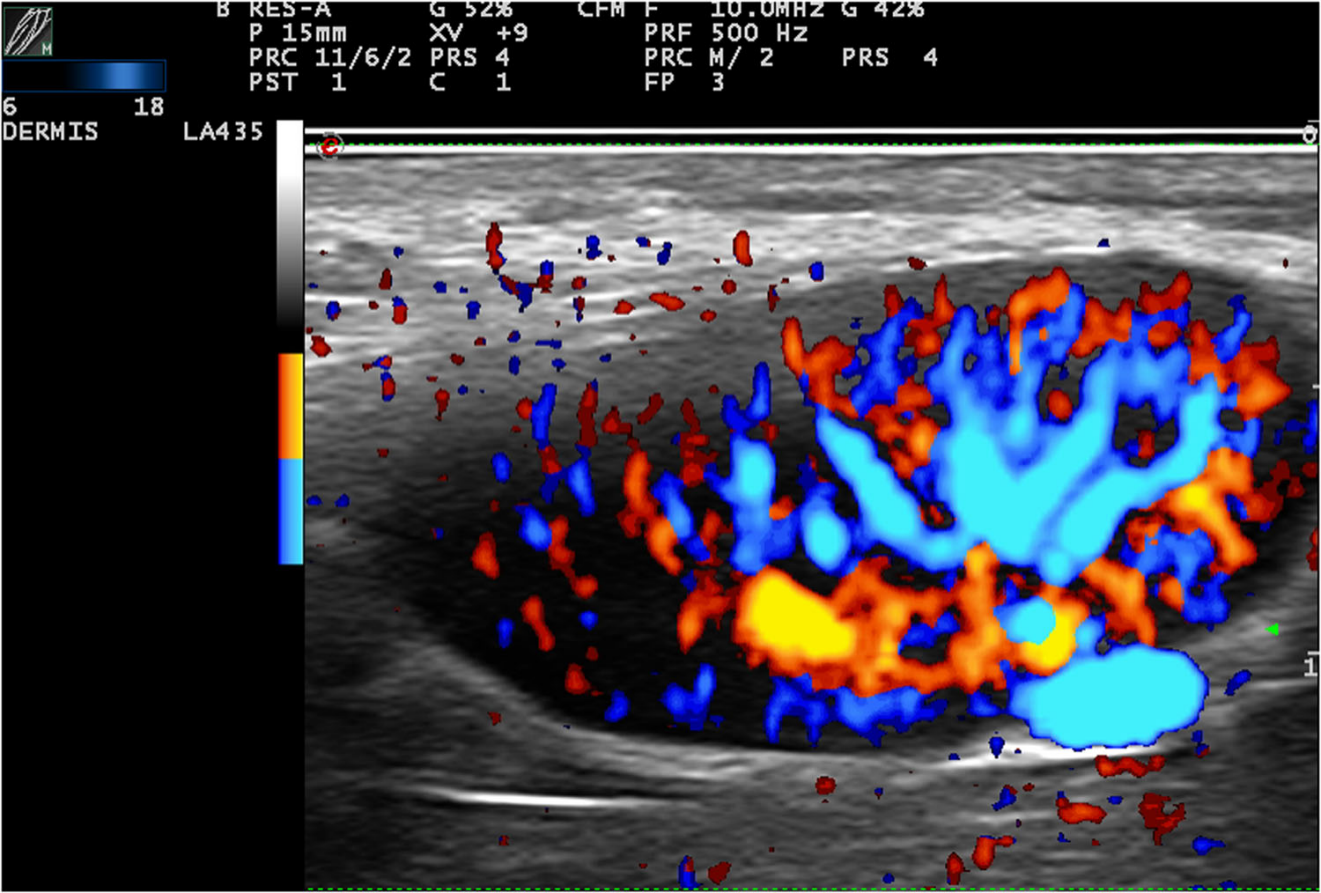
Case 3. High‐frequency ultrasound pattern of inflammation in an enlarged lymph node.

In one patient, an enlarged axillary lymph node was observed on the same side of the vaccine application. The node measured 20.1 x 9.3 mm with intense vascularisation distributed from the hilum towards the periphery, had arterial vessels up to 1.2 mm in diameter and a very high velocity (a systolic peak of 37.2 cm/s and resistance index of 0.87) (Figure 7). A characteristic HFUS pattern of inflammation was observed in this patient, with increased flow from the hilum to the periphery.

## DISCUSSION

We observed that patients with COVID‐Arm had inflammation of the dermis and the hypodermis, with signs of panniculitis, similar to those seen with HFUS in autoimmune and infectious diseases.^10^ The colour Doppler mode allowed us to quantify the inflammation and indicate treatment according to clinical and ultrasonographic signs. In addition, other findings such as inflammatory lymph nodes were observed.

COVID‐Arm is usually diagnosed on clinical grounds.^1^ In most cases, inflammation resolves spontaneously or with topical anti‐inflammatory treatment after a few days.^2^ Therefore, skin biopsy is usually not necessary. Some patients may have persistent lesions that require longer topical treatment or even oral corticosteroid therapy.

HFUS is a technique that has become widespread in a routine dermatology practice in recent years.^10^ It can be carried out at the office and aids in therapeutic decision making, by comparing clinical and imaging findings. In inflammatory diseases, HFUS is helpful to define the site of involvement (whether dermal, hypodermic, or both) and quantify vascularisation.^8^, ^10^ In panniculitis, HFUS has proven helpful in assessing lobular, septal, or mixed patterns. Finally, HFUS permits assessment of response to treatment during follow‐up.

In our case series, COVID‐Arm affected the deeper layers of the skin. This was demonstrated with HFUS, without the need for a skin biopsy. We propose that HFUS is a useful noninvasive imaging technique for a complete assessment of vaccine reactions.

## CONFLICT OF INTEREST

The authors declare no conflict of interest.

## ETHICS STATEMENT

The study was authorized by the Ethics Committees of our institution. All patients gave written informed consent to participate and explicit consent to publish images.

## Data Availability

Materials, data, and associated protocols are available upon request to readers.

## References

[1] Català A , Muñoz‐Santos C , Galván‐Casas C , Roncero Riesco M , Revilla Nebreda D , Solá‐Truyols A , et al. Cutaneous reactions after SARS‐COV‐2 vaccination: a cross‐sectional Spanish nationwide study of 405 cases. Br J Dermatol. 2022;186:142–52. 34254291 10.1111/bjd.20639 PMC8444756

[2] Fernandez‐Nieto D , Hammerle J , Fernandez‐Escribano M , Moreno‐del Real CM , Garcia‐Abellas P , Carretero‐Barrio I , et al. Skin manifestations of the BNT162b2 mRNA COVID‐19 vaccine in healthcare workers. ‘COVID‐arm’: a clinical and histological characterization. J Eur Acad Dermatol Venereol. 2021;35:e425–7. 33783873 10.1111/jdv.17250 PMC8251500

[3] Gregoriou S , Kleidona IA , Tsimpidakis A , Nicolaidou E , Stratigos A , Rigopoulos D . ‘COVID vaccine arm’ may present after both mRNA vaccines vaccination. J Eur Acad Dermatol Venereol. 2021;35:e867–8. 34416053 10.1111/jdv.17614 PMC8657342

[4] Vaccaro M , Bertino L , Squeri R , Genovese C , Isola S , Spatari G , et al. Early atypical injection‐site reactions to COVID‐19 vaccine: a case series. J Eur Acad Dermatol Venereol. 2022;36:e24–6. 34547113 10.1111/jdv.17683 PMC8656410

[5] Wei N , Fishman M , Wattenberg D , Gordon M , Lebwohl M . “COVID arm”: a reaction to the moderna vaccine. JAAD Case Rep. 2021;10:92–5. 33748377 10.1016/j.jdcr.2021.02.014 PMC7959672

[6] Blumenthal KG , Freeman EE , Saff RR , Robinson LB , Wolfson AR , Foreman RK , et al. Delayed large local reactions to mRNA‐1273 vaccine against SARS‐CoV‐2. N Engl J Med. 2021;384:1273–7. 33657292 10.1056/NEJMc2102131 PMC7944952

[7] Chen LF , Lu CH , Shen CY , Hsieh SC , Li KJ . Sonographic findings of cutaneous panniculitis in systemic lupus erythematosus and/or antiphospholipid syndrome: a case series. Ultrasound Med Biol. 2017;43:S196–7.

[8] Marti‐Marti I , Morgado‐Carrasco D , Podlipnik S , Rizo‐Potau D , Bosch‐Amate X , Lledó GM , et al. Usefulness of high‐frequency ultrasonography in the evaluation and monitoring of sclerosing dermatoses: a cohort study. Clin Exp Dermatol. 2022;47:351–8. 34431556 10.1111/ced.14903

[9] Baden LR , El Sahly HM , Essink B , Kotloff K , Frey S , Novak R , et al. Efficacy and safety of the mRNA‐1273 SARS‐CoV‐2 vaccine. N Engl J Med. 2021;384:403–16. 33378609 10.1056/NEJMoa2035389 PMC7787219

[10] Wortsman X , Wortsman J , Sazunic I , Carreño L . Activity assessment in morphea using color Doppler ultrasound. J Am Acad Dermatol. 2011;65:942–8. 21550692 10.1016/j.jaad.2010.08.027

